# Prognostic factors in patients who underwent surgery for common peroneal nerve injury: a nest case–control study

**DOI:** 10.1186/s12893-020-01033-x

**Published:** 2021-01-06

**Authors:** Zhenhui Liu, Maimaiaili Yushan, Yanshi Liu, Aihemaitijiang Yusufu

**Affiliations:** Department of Microrepair and Reconstruction, The First Affiliated Hospital of Xinjiang Medical University, Urumqi, Xinjiang People’s Republic of China

**Keywords:** Common peroneal nerve injury, Nest case–control study, Prognostic risk factors

## Abstract

**Background:**

Common peroneal nerve (CPN) injury is one of the most common nerve injuries in the lower extremities and the motor functional recovery of injured common peroneal nerve (CPN) was often unsatisfactory, the mechanism of which is still controversial. The purpose of this retrospective study was to determine the prognostic factors in patients who underwent surgery for CPN injury and provide a tool for clinicians to assess the patients’ prognosis.

**Methods:**

This is a retrospective cohort study of all patients who underwent neural exploration for injured CPN from 2009 to 2019. A total of 387 patients with postoperative follow-up more than 12 months were included in the final analysis. We used univariate logistics regression analyses to explore explanatory variables which were associated with recovery of neurological function. By applying multivariable logistic regression analysis, we determined variables incorporated into clinical prediction model, developed a nomogram by the selected variables, and then assessed discrimination of the model by the area under the curve (AUC) of the receiver operating characteristic (ROC) curve.

**Results:**

The case group included 67 patients and the control group 320 patients. Multivariate logistic regression analysis showed that area (urban vs rural, OR = 3.35), occupation(“blue trouser” worker vs “white-trouser” worker, OR = 4.39), diabetes (OR = 11.68), cardiovascular disease (OR = 51.35), knee joint dislocation (OR = 14.91), proximal fibula fracture (OR = 3.32), tibial plateau fracture (OR = 9.21), vascular injury (OR = 5.37) and hip arthroplasty (OR = 75.96) injury increased the risk of poor motor functional recovery of injured CPN, while high preoperative muscle strength (OR = 0.18) and postoperative knee joint immobilization (OR = 0.11) decreased this risk of injured CPN. AUC of the nomogram was 0.904 and 95% CI was 0.863–0.946.

**Conclusions:**

Area, occupation, diabetes, cardiovascular disease, knee joint dislocation, proximal fibula fracture, tibial plateau fracture, vascular injury and hip arthroplasty injury are independent risk factors of motor functional recovery of injured CPN, while high preoperative muscle strength and postoperative knee joint immobilization are protective factors of motor functional recovery of injured CPN. The prediction nomogram can provide a tool for clinicians to assess the prognosis of injured CPN.

## Background

Common peroneal nerve (CPN) injury is one of the most common nerve injuries in the lower extremities, which can lead to loss of sensation of the anterolateral foot, and/or a foot drop and result in gait disturbances followed by serious consequences for patients who were not treated properly. Attributed to various factors, including its internal organization, blood supply, superficial topography over the fibular head, and its location, CPN seems particularly prone to injury from iatrogenic accidents, motor vehicle accidents, sport injuries, and gunshot wounds [1–4]. Compression and entrapment lesions are probably the most frequent causes of peroneal neuropathy [5]. The CPN may be compressed by a ganglion cyst, cysts of lateral meniscus, or a tumor of the head of the fibula.

Although the regeneration ability of the peripheral nerve is stronger than that of the central nerve system and the function of peripheral nerve can be recovered to a certain extent, the injured peripheral nerve cannot recover under some circumstances [6, 7]. However, there are significant differences in prognosis of different peripheral nerve injuries. While previously published papers showed encouraging clinical results [1, 8–10], some recent studies showed pessimistic results with CPN injuries. Compared with injured tibial nerve, the functional recovery of injured CPN was often unsatisfactory, the mechanism of which was still not clear [11–16]. Terzis showed that associated fractures and/or vascular injury, the mechanism and type of injury, denervation time, nerve gap and graft length, and the surgical strategies might affect the functional outcome of CPN [17]. Nevertheless, the factors influencing the prognosis of CPN were controversial.

The purpose of this retrospective study was to determine the factors associated with the motor functional recovery of injured CPN in patients who underwent surgery for CPN injury and to develop a tool for clinicians to assess the patients’ prognosis.

## Methods

Patients who underwent neural exploration of injured CPN from January 2009 to January 2019 were included in this retrospective cohort study which was approved by the Ethics Committee of our institution. All the subjects had signed the informed consent form before surgery. Inclusion criteria consisted of (1) patients with open CPN injuries, (2) patients with closed fracture who had clinical symptoms of CPN injury and underwent surgical exploration during the treatment of fracture, (3) patients with definitive CPN injuries without improvement after 3 months of conservative treatment, and (4) patients with follow-up period more than 12 months after surgery. Exclusion criteria were constitutive of (1) patients with incomplete medical record, (2) patients with follow-up period less than 12 months or loss of follow up after treatment, (3) patients with diabetic peripheral neuropathy, (4) patients presented with foot drop caused by central nerve disease, (5) patients with definitive CPN injuries without motor dysfunction, and (6) patients underwent ankle arthrodesis or amputation due to severe trauma.

**Fig. 1:**
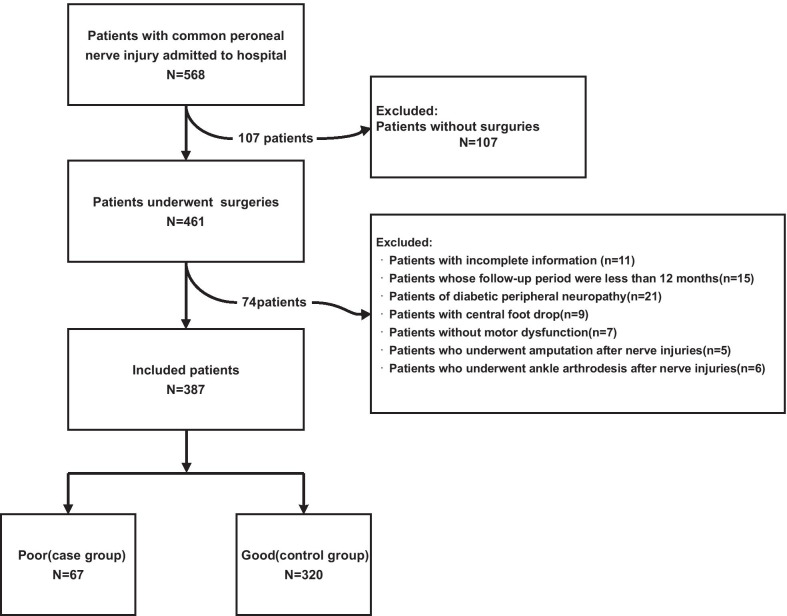
Flowchart of participants

A total of 568 patients with injured CPN were admitted in our hospital from January 2009 to January 2019, of which 181 cases were excluded from the study based on our exclusion criteria. The remaining 387 patients were divided into control group with good result (n = 320) and case group with poor result (n = 67) according to the BMRC grading system and the last follow-up evaluation [18, 19] (Fig. 1).

The basic information of patients was collected, including age, sex, area (urban and rural), occupation (“white-trouser” worker and “blue-trouser” worker), educational background (low and high level), medical history (diabetes, hypertension and cardiovascular disease), drinking history, smoking history, weight and height. We distinguished urban areas (municipalities and prefecture-level cities) from rural areas (county-level cities, towns and villages) according to administrative districts. We defined workers who require intense lower extremities use as “blue trouser” workers, such as individuals in building and grounds maintenance, construction and extraction, food preparation and serving, and transportation and material moving occupations. The workers who required very limited lower extremities use were defined as “white-trouser” workers. Patients with 12 years or more of formal education, including college and postgraduate education, were defined as high education level, while the others with 11 years or fewer, including elementary, high school education and lower secondary education, were defined as low education level. The formula (BMI = weight (kg) /height (m^2^)) was used to calculate the body mass index (BMI) ^20^. Patients with BMI lower than 18.5 were defined as thin, between 18.5 < BMI < 24 kg/m^2^ as normal, BMI between 24 and 28 kg/m^2^ as overweight, and BMI ≥ 28 kg/m^2^ as obesity.

Factors related to the CPN injuries were also collected which included the injured side, etiology of injuries, duration of symptoms and innervated muscle strength. Etiology of injury [5, 21, 22] consisted of (1) scar formation, including skin scar and posttraumatic scar formed by connective tissue, (2) knee injuries, including direct or indirect trauma, open injuries, knee dislocation and fracture of the fibular head and tibial plateau, (3) anatomic factors, which caused secondary entrapment due to a fibrous band at the origin of the peroneus longus, (4) external compression sources, for example, tight splint/cast and compression wrapping/bandage, (5) iatrogenic injury from hip arthroplasty injury or knee arthroplasty injury, (6) hip fracture, including acetabular fracture, femoral neck fractures and intertrochanteric fracture, and (7) vascular injury, caused by femoral artery embolization or popliteal embolization.

Laboratory results on the day of admission were collected and analyzed. White blood cells (WBC), red blood cells (RBC), hemoglobin (HGB), and platelets (PLT) were performed by an automated hematology analyzer (SYSMEX 2000; Sysmex Corp., Kobe, Japan). Total protein (TP) and albumin (ALB), triglyceride (TG), total cholesterol (TC), high-density lipoprotein cholesterol (HDL-c), low-density lipoprotein cholesterol (LDL-c), apolipoproteins A1(Apoa1), apolipoproteins B (Apob), blood glucose (Glu), serum potassium ion (K), serum sodium (Na), serum calcium (Ca), serum phosphorus (P) and serum magnesium (Mg) were measured by the Dimension AR/AVL Clinical Chemistry System (Newark, NJ, USA) and its supporting reagents. All of tests were operated in the clinical laboratory of our institution.

The CPN was explored under the operating microscope. According to the intraoperative findings, the external compression factors causing the nerve entrapment were released completely. Patients with severe adhesion among nerve bundles caused by scar or hematoma were treated with endoneurolysis. Patients with obvious edematous nerve, blurred or disappeared neurovascular network were treated with epineural neurolysis. The released peroneal nerve was placed in a soft tissue bed with enough blood supply. The neuroma was resected and the nerve was repaired by end to end suture. Soleus muscle branch of the tibial nerve was transfer to the distal CPN when nerve defect could not be directly sutured even under joint flexion position. Patients with atrophy of anterior tibial muscle or extensor digitorum longus muscle were treated with posterior tibial tendon transfer (PTTF) at the same time of neurolysis. Patients with shortened Achilles tendon malformation, were treated with Achilles tendon lengthening at the same time of neurolysis. According to classification of nerve injuries described by Sunderland [18, 19], the injured nerves was divided into five types in line with the intraoperative observation.

Plaster immobilization of the knee joint was performed in patients with unstable fracture, nerve by end-to-end suture or soleus muscle branch transfer for 4 weeks after operation. And, plaster fixation in dorsal extension of ankle was performed in patients with Achilles tendon lengthening or PTTF. All the patients were treated with oral vitamin B for 3 months and underwent physiotherapy.

Muscular strength of ankle dorsiflexion and toe dorsal extension was assessed according to the British Medical Research Council (BMRC) scoring system at patient’s last visit. The clinical outcomes were categorized as poor (case group) if the muscular strength of ankle dorsiflexion or toe extension ≤ M2, and as good (control group) if the muscular strength ankle dorsiflexion and toe extension ≥ M3 [14].

### Statistical analysis

The collected data were independently collected, verified and corrected by two staff members using EpiData 3.1 software (EpiData Association, Odense, Denmark). Statistical analyses were implemented by R Studio (Version 1.2.5001) with rms, ROCR, gplots and forestplot packages. Variables included were tested for normality, and the skewed distribution variables were transformed by natural logarithm. The continuous variables, analyzed using the Student *t* test, were expressed as mean ± standard deviation (SD), and the count variables, detected using the Chi-square or Fisher’s test, were expressed as number (%). Two-tailed analysis with p-value < 0.05 was considered as statistically significant level.

Taking outcomes as bivariate dependent variables and the other factors as independent variables, we used univariate logistics regression analyses to assess which explanatory variables are associated with recovery. Variables with statistical significance were fitted to regression model 1. We incorporated the variables of p-value (< 0.1) in univariate analysis into multivariate logistic regression analysis by stepwise method. The variables with low contribution (p-value < 0.05) to the model were eliminated by stepwise selection method. Reserved variables were fitted to regression model 2. Better model selected by sensitivity analysis was used to develop the nomogram. The neural function recovery discrimination was assessed by the area under the curve (AUC) of the receiver operating characteristic (ROC) curve.

## Results

### Patients

Three hundred eighty-seven patients with injured CPN were included in the final analysis. The case group consisted of 67 (17.31%) patients with a mean age of (43.03 ± 15.73) years and a mean BMI of (23.55 ± 4.30) kg/m^2^, while the control group consisted of 320 (82.69%) patients with a mean age of (33.41 ± 14.52) years and a mean BMI of (23.32 ± 4.06) kg/m^2^. Patients with age of (20–29) years and normal BMI had highest percentage in both case group and control group. However, there were no statistical difference of component percentage of age subgroups and BMI subgroups between case group and control group. Approximately 74.63% of case group and 76.25% of control group were male. The percentage of patients from rural areas in case group (23.88%) was lower than that in control group (53.44%), and the percentage of patients engaged in manual work in case group (35.82%) was lower than that in control group (49.69%). The constituent ratios of patients with smoking history, drinking history and hypertension history in case group and control group had no statistical difference. Approximately 13.43% of case group and 1.88% of control group had type 2 Diabetes history. Compared with control group, case group had a higher rate (13.43%) with cardiovascular disease. (Table 1).

**Table 1:** Socio-demographic characteristics of patients in case and control group

Variable	Total (n = 387)	Case group (n = 67)	Control group (n = 320)	P-value
Age (years)**	35.07 ± 15.16	43.03 ± 15.73	33.41 ± 14.52	< 0.001
0 ~	63 (16.28)	1 (1.49)	62 (19.38)	0.263
20 ~	91 (23.51)	24 (35.82)	67 (20.94)	
30 ~	83 (21.45)	16 (23.88)	67 (20.94)	
40 ~	71 (18.35)	11 (16.42)	60 (18.75)	
50 ~	53 (13.70)	11 (16.42)	42 (13.12)	
60 ~	26 (6.72)	4 (5.97)	22 (6.87)	
Sex				0.777
Male	294 (75.97)	50 (74.63)	244 (76.25)	
Female	93 (24.03)	17 (25.37)	76 (23.75)	
Areas**				< 0.001
Rural	187 (48.32)	16 (23.88)	171 (53.44)	
Urban	200 (51.68)	51 (76.12)	149 (46.56)	
Occupation*				0.039
“White-trouser” workers	183 (47.29)	24 (35.82)	159 (49.69)	
“Blue-trouser” workers	204 (52.71)	43 (64.18)	161 (50.31)	
Education				0.614
High	78 (20.16)	12 (17.91)	66 (20.63)	
Low	309 (79.84)	55 (82.09)	254 (79.37)	
Smoking	42 (10.85)	4 (5.97)	38 (11.88)	0.158
Drinking	4 (1.03)	3 (4.48)	1 (0.031)	0.131
Type 2 Diabetes**	12 (3.10)	9 (13.43)	3 (0.94)	< 0.001
Hypertension	9 (2.33)	3 (4.48)	6 (1.88)	0.401
Cardiovascular disease **	10 (2.58)	9 (13.43)	1 (0.031)	< 0.001
BMI	23.36 ± 4.06	23.55 ± 4.30	23.32 ± 4.06	0.677
Thin	34 (8.79)	8 (11.94)	26 (8.12)	0.626
Normal	198 (51.16)	28 (41.79)	170 (53.13)	
Overweight	119 (30.75)	24 (35.82)	95 (29.69)	
Obesity	36 (9.30)	7 (10.45)	29 (9.06)	
Surgery **				< 0.001
End-to-end suture	48 (12.40)	2 (2.99)	46 (14.36)	
Neurolysis	286 (73.90)	63 (94.03)	63 (94.03)	
Nerve transfers and neurolysis	13 (3.36)	2 (2.99)	11 (3.44)	
PTTF and neurolysis	30 (7.75)	0 (0)	30 (9.37)	
Achilles tendon lengthening and neurolysis	10 (2.58)	0 (0)	10 (3.14)	
Post operation				
Follow-up time (months)	15.29 ± 2.60	14.67 ± 2.81	15.37 ± 1.93	0.791
Knee Immobilization **	122 (31.52)	6 (8.96)	(36.25)	< 0.001

Values presented as mean ± SD or frequencies and percentages, n (%). *P < 0.05, **P < 0.01

*P < 0.05, **P < 0.01

Table 2 presented factors associated with CPN injury. Case group had significantly higher rate of tibial plateau fracture, knee dislocation, fracture of the proximal fibula anatomic factors, hip arthroplasty injury, knee arthroplasty injury, hip fracture, and vascular injury, while control group had higher rate of myodynamia. Type of nerve injury on basis of Sunderland type had statistical difference between case group and control group. The details were shown on Table 2. Compared with controls, the cases had higher TP, Glu and lower Na, Ca (P < 0.05). There were no significant differences in WBC, RBC, HGB, PLT, ALB, TG, TC, HDL-c, Apoa, Apob, K, P and Mg between the case group and control group. The differences of treatment characteristics between case and control group shown on Table 1. Constituent ratio of surgery type had statistical difference between case group and control group. There was no statistical difference of follow-up time between case group and control group. Compared with case group, control group had higher knee immobilization rate.

**Table 2:** Common peroneal nerve injuries characteristics of patients in case and control group

Variables	Total (n = 387)	Case group (n = 67)	Control group (n = 320)	P-value
Side				0.989
Left	214 (53.77)	37 (55.22)	177 (55.31)	
Right	173 (43.47)	30 (44.78)	143 (44.68)	
Duration (months)	11.43 ± 21.18	12.42 ± 22.74	11.23 ± 20.97	0.519
Scar compression				0.739
No	195 (48.99)	35 (52.24)	160 (50.00)	
Yes	192 (48.24)	32 (47.76)	160 (50.00)	
knee injury				
Tibial plateau fracture**	16 (4.02)	8 (11.94)	8 (2.50)	0.001
Knee dislocation*	16 (5.00)	12 (3.10)	4 (5.97)	< 0.001
Direct injury	187 (46.98)	30 (44.78)	157 (49.06)	0.523
Fracture of the proximal fibula*	90 (23.26)	30 (44.78)	60 (18.75)	< 0.001
Anatomic factors*				0.039
No	356 (89.45)	64 (95.52)	12 (3.75)	0.495
Yes	31 (7.79)	3 (44.78)	4 (0.94)	0.466
External compression				
Plint/cast	16 (4.13)	4 (5.97)	12 (3.75)	0.495
Wrapping/bandage	4 (1.03)	0 (0.00)	4 (1.25)	0.466
Iatrogenic injury				
Hip arthroplasty injury**	7 (1.81)	6 (8.96)	1 (0.31)	< 0.001
Knee arthroplasty injury**	13 (3.40)	8 (11.94)	5 (1.56)	< 0.001
Hip fracture**	9 (2.26)	5 (7.46)	4 (1.25)	0.009
Vascular injury**	22 (5.68)	15 (2.39)	7 (2.19)	< 0.001
Muscle strength**	233 (60.21)	27 (40.30)	206 (64.38)	< 0.001
Sunderland classification				< 0.001
I	73 (18.86)	15 (22.39)	58 (18.13)	
II	86 (22.22)	3 (4.48)	83 (25.94)	
III	135 (34.88)	37 (55.22)	37 (55.22)	
IV	27 (6.98)	18 (5.63)	9 (13.43)	
V	66 (17.05)	3 (4.48)	63 (19.69)	

Values presented as mean ± SD or frequencies and percentages, n (%)

*P < 0.05, **P < 0.01

### Univariate logistic regression analysis

**Fig. 2:**
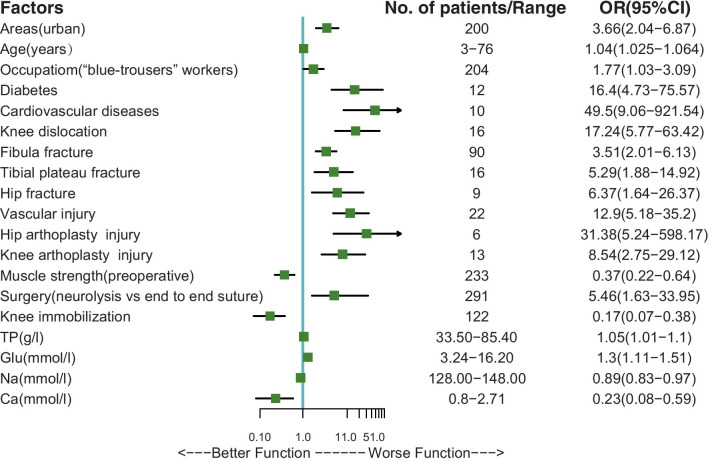
Results of univariate logistic regression analysis

In Univariate logistic regression analysis, area(urban vs rural), age, occupation(“blue trouser” workers vs “white-trouser” workers), T2 diabetes, cardiovascular disease, knee joint dislocation, proximal fibula fracture, tibial plateau fracture, hip fracture, vascular injury, hip joint arthroplasty injury, knee joint arthroplasty injury, surgery (neurolysis vs end to end suture), high total protein concentration and high blood glucose concentration increased the risk of poor motor functional recovery of injured CPN. High preoperative muscle strength, knee immobilization, high serum calcium concentration, and serum sodium concentration reduce this risk of CPN (Fig. 2). Those variates with statistical difference (p < 0.05) were fitted into model 1, while the others were not included in the subsequent analysis.

### Multivariate logistic regression analysis

**Fig. 3:**
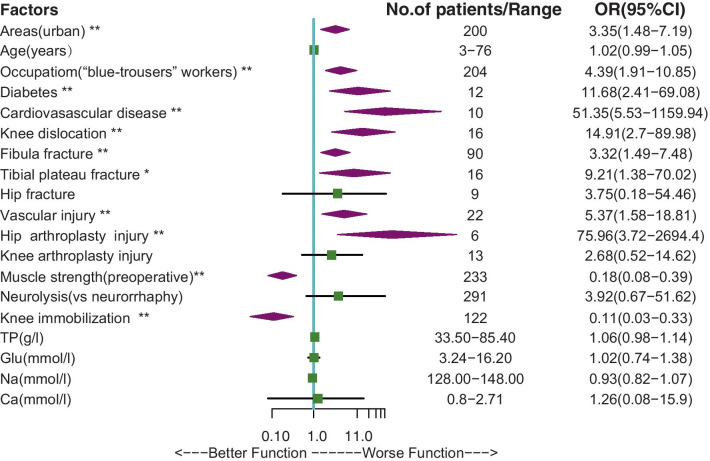
Results of multivariate logistic regression analysis

The multivariate logistic regression analysis showed that, area (urban vs rural, OR = 3.35, 95% CI 1.48–7.19), occupation (“blue trouser” workers vs “white-trouser” workers, OR = 4.39, 95% CI 1.91–10.85), diabetes (OR = 11.68, 95% CI 2.41–69.08), cardiovascular disease (OR = 51.35, 95% CI 5.53–1159.94), knee joint dislocation (OR = 14.91, 95% CI 2.7–89.8), proximal fibula fracture (OR = 3.32, 95% CI 1.49–7.48), tibial plateau fracture (OR = 9.21, 95% CI 1.38–70.02), vascular injury (OR = 5.37, CI 1.58–18.81) and hip arthroplasty (OR = 75.96, 95% CI 3.72–2694.40) injury increased the risk of poor motor functional recovery of injured CPN, while high preoperative muscle strength (OR = 0.18, 95% CI 0.08–0.39) and postoperative knee joint immobilization (OR = 0.11, 95% CI 0.03–0.33) decreased this risk of CPN (Fig. 3). We fitted those variates of statistical difference (P< 0.05) into model 2.

### Establishment of clinical prediction nomogram

**Fig. 4:**
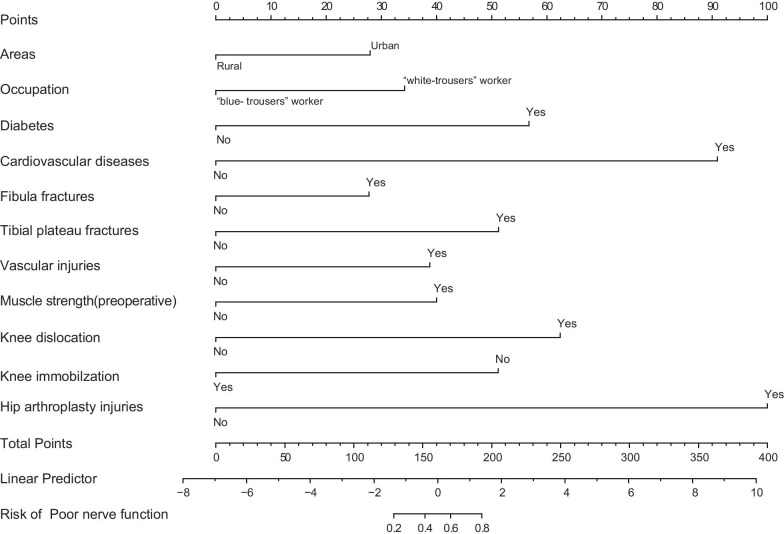
Nomogram to predict the probability of poor nerve function in the patient with common peroneal

**Fig. 5:**
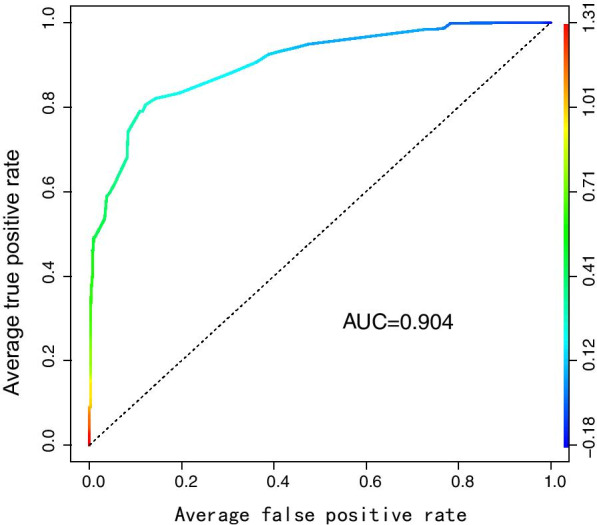
ROC curves for validating the discrimination power of the nomogram

The sensitivity of model 1 and model 2 was analyzed by chi-square test. The results (P = 0.27) showed that the increased variables (age, hip fracture, knee arthroplasty injury, total protein concentration, blood glucose concentration, blood sodium concentration, blood calcium concentration) in model 1did not increase the accuracy of the model, so we used the simpler model 2 to construct clinical predictive model and the nomogram (Fig. 4). AUC of this predictive model was 0.904, and the 95% CI was 0.863–0.946 (Fig. 5).

## Discussion

In this study, we found that area(rural), occupation(“blue trouser” worker), diabetes, cardiovascular disease, knee joint dislocation, proximal fibula fracture, tibial plateau fracture, vascular injury and hip arthroplasty injury are independent risk factors of motor functional recovery of CPN, while high preoperative muscle strength and postoperative knee joint immobilization are protective factor of motor functional recovery of CPN. Using these factors, we developed a nomogram which could likely predict the level of common peroneal nerve motor recovery after surgery.

The effect of the blood supply to the CPN on prognosis was highlighted after multifactor logistic regression analysis. Reports had found the prognosis of injured CPN at the thigh level was slightly better than that at the hip area [16]. In recent years, a growing number of reports found that the peroneal nerve of the sciatic nerve was more vulnerable and difficult to recover [4, 14, 17]. We also found CPN injuries after hip arthroplasty had better prognosis than that of knee arthroplasty. It was not clear whether this was due to more severe damage to the CPN because of its anatomical location or other factors [15, 21]. Blood supply of CPN had drawn attention of researchers [21, 23–26]. The nutrient arteries of the peripheral nerves are anatomically located in the connective tissue sheath, the nerve bundles and inside the nerve fibers which guarantee sufficient blood supply in cases that the vessels are interrupted [27]. The extraneural arterial chain of the sciatic nerve consist of 2–6 nutritive arteries at certain intervals which originated from inferior gluteal artery and popliteal artery branches[28]. The extraneural arterial chain of the tibial nerve was supported by 2–5 nutritive arteries formed by branch of the tibiofibular trunk, peroneal artery, and posterior tibial artery[28]. Thus, one or more of those nutritive arteries interruption had no significant effect on the blood of sciatic nerve and tibial nerve. However, the part of the CPN from the terminal division of the sciatic nerve to the fibular neck were supplied by a single blood vessel (97.2%) [26]. Hence, the CPN tended to have a poor prognosis after injuries with the necrosis of nerve ischemic edema, Wallerian degeneration, and the formation of fibrous scar [14, 16].

We believed that knee joint dislocation, proximal fibula fracture, tibial plateau fracture and hip arthroplasty injury affect the functional recovery because of limited blood supply interruption rather than nerve fiber damage. We also observed that vascular injury diseases, including femoral and popliteal artery embolization, were a risk factor both in univariate and multifactorial analyses, which could be explained by vascular thrombosis or embolism of the CPN associated with inadequate collateral circulation [25, 26].

The fact that diabetic patients were vulnerable to peripheral nerve damage had been reached an agreement [29, 30]. Diabetic tended to develop complications of neuropathy or vasculopathy. An underlying neurologic disease increased the problems for any patient with a nerve injury. Cardiovascular disease and diabetes had been found to increase the incidence of CPN in cardiothoracic operations [23]. Patients with cardiovascular disease may have systemic vascular sclerosis, so that blood supply was not easy to restore after nerve injury. Nerve regeneration of injured nerve in diabetic patients was often impaired because of microangiopathic involvement of the vasa nervorum [31]. The fact that patients with cardiovascular disease or diabetes tended to have poor prognosis in our research, indicated that microcirculation of CPN played an ignored role in the repair of CPN.

We found few reports about the influence of “blue trouser” workers on the recovery of the CPN. Excessive exercise or positional factors of “blue trouser” workers could result in chronic compartment at the neck of the fibula, causing subclinical neuropathy of CPN [32],which might have potential influence on the recovery of nerve function after injury. Furthermore, compared with “white-trouser” workers, “blue trouser” workers might have stronger tibialis posterior muscle, so that limited recovery of CPN function could not confront the tibialis posterior muscle. There were few studies on the reason why patients living in urban areas had poor neurological prognosis. Patients from rural areas tended to have good prognosis, which could be related to more increased physical activities because they were in a less populated area. However, living in the urban area was not the cause of failure or surgery, but rather the likelihood of specific injury was more possible. Thus, those factors needed further investigation.

Our research found that high preoperative muscle strength, and postoperative knee joint immobilization decreased the risk of poor recovery of CPN injury. The prognosis of patients was better with higher preoperative muscle strength, because residual innervation could avoid muscle atrophy, thus enabling better recovery of reinnervation. Knee joint immobilization flexion position could avoid the repeated stimulation of swollen nerves, reduce the probability of vascular occlusion and the duration and degree of edema, and shorten the time of ischemia.

Obesity seemed to have higher complication rates related to nerve surgery [33, 34]. However, fat pad surrounding the fibular head could protect the CPN [35]. Our research suggested that obese patients with higher prevalence of neural injuries did not have worse prognosis compared with normal patients. We also found no difference of neural functional recovery among patient with different BMI. The adverse effects of smoking was found on the functional recovery of peripheral nerves after ischemia/reperfusion injuries in rats [36]. Whereas, a meta-analysis on prognostic associations of peroneal nerve decompression found that although smoking increased the trend of pain, outcomes were not affected by presentation [37]. In our study, smoking had no impaction on the recovery of CPN injuries.

Age and sex showed no correlation with prognosis after surgeries in present studies. A review [37] showed that outcomes did not vary with an advanced age or sex after peroneal nerve decompression. Another review [6], which included 28 studies to assess the results of repaired CPN, found no significant relationship between outcome and patient age. Our analysis similarly showed that neither age nor sex were prognostic factors of repaired CPN.

Univariate logistic regression analysis showed that the surgical type of neurolysis yielded a worse outcome than end to end suture. Nevertheless, after multifactor logistic regression analysis, there were no statistical difference in outcome of repaired CPN among different surgical types, which was consistent with previous reports [6, 37–39]. Among the variables which could affect the function recovery of repaired CPN, mechanism of injury was one of the crucial determining factors. In experimental nerve injuries, function of injured nerve caused by a sharp transection was easier to restore, compared with that by an avulsion [38]. The avulsed nerves showed no normal nerve architecture at any time period, while the cut specimens showed a progressive resolution in the zone of injury. Prasad et al. [39] insisted that stretch/traction injury zone extended into the myoneural junction, creating scar tissue that prevented motor reinnervation, which was the reason of poor functional motor recovery after reconstruction of traction injury to the CPN. However, all the surgical types could not achieve reinnervation of the peroneal innervated muscles because the traction injuries extending beyond what can be perceived optically, even with operating microscope. Besides, stretch/traction injury of CPN provided another perspective on the poor motor function of injured CPN caused or accompanied by knee joint dislocation, proximal fibula fracture, tibial plateau fracture and hip arthroplasty injury in our research.

There were perspectives that tendon transfer should be added in all patients at the time of nerve reconstruction [40]. However, additional surgeries led to greater trauma and increased the risk of uncertain complications, including infection, overcorrection, instability, rupture of tendon transfer and cocked-up hallux [41]. Our research suggested that though traction lesions could be extensive, the prognosis seemed to vary considerably. Therefore, both clinicians and patients need quantified indicators to estimate the prognosis of injured CPN without additional surgeries in early stage of treatment.

To our knowledge, this study is the first to assess factors associated with injured CPN and establish a prediction model to predict the prognosis of injured CPN by using a nomogram. It is generally believed that the model with AUC of 0.50–0.75 is acceptable, and AUC > 0.75 indicates that the discrimination of model is prominent [42]. AUC of our prediction model is 0.904, so this nomogram can be used to predict the prognosis of injured CPN well. Our study was carried out in patients with high-risk of poor prognosis, which could improve the efficiency of model for risk factors. Besides, selected factors used to construct prediction model are relatively objective, which is helpful for further application of this model. Using the nomogram, a clinician can eyeball the sum of all predictors’ effect for a given patient with injured CPN, and predict the prognosis of injured CPN. The nomogram could provide evidence for clinicians to assess whether a patient need aggressive surgical strategies in the early treatment stage of injured CPN, such as tendon transfer, ankle foot orthosis, or arthrodesis.

There are limitations of our study that are notable. First, this study was limited as a monocentric analysis. Although there are a lot of cases, we still need evidence from other centers to verify this model. In subsequent research work, therefore, we will persuade other medical center to join in this research project, and provide the corresponding clinical data for further evaluation and validation of the prediction model. Second, our cohort was limited to patients with injured CPN and requirement of surgical treatment.

## Conclusions

We found that area, occupation, diabetes, cardiovascular disease, knee joint dislocation, proximal fibula fracture, tibial plateau fracture, vascular injury and hip arthroplasty injury are independent risk factors of motor functional recovery of CPN, while high preoperative muscle strength and postoperative knee joint immobilization are protective factor of motor functional recovery of CPN. The blood supply and stretch/traction injury to the CPN is significant factors worth being paid more attention to. The prediction nomogram can provide a tool for clinicians to assess the prognosis of injured CPN.

## Data Availability

All datasets used and/or analyzed during the current study are available from the corresponding author on reasonable request.

## Abbreviations

ALB: Albumin
Apoa1: Apolipoproteins A1
Apob: Apolipoproteins B
AUC: Area under the curve
BMI: Body mass index
BMRC: British Medical Research Council
Ca: Serum calcium
CI: Confidence intervals
CPN: Common peroneal nerve
Glu: Blood glucose
HGB: Hemoglobin
HDL-c: High-density lipoprotein cholesterol
K: Serum potassium ion
LDL-c: Low-density lipoprotein cholesterol
Mg: Serum magnesium
Na: Serum sodium
P: Serum phosphorus
PLT: Platelets
RBC: Red blood cells
ROC: Receiver operating characteristic
SD: Standard deviation
TC: Total cholesterol
TG: Triglyceride
TP: Total protein
WBC: White blood cells
PTTF: Posterior tibial tendon transfer
